# Protective effects of melatonin against the toxic effects of environmental pollutants and heavy metals on testicular tissue: A systematic review and meta-analysis of animal studies

**DOI:** 10.3389/fendo.2023.1119553

**Published:** 2023-01-30

**Authors:** Niloofar Dehdari Ebrahimi, Shima Parsa, Farnoosh Nozari, Mohammad Amin Shahlaee, Amirhossein Maktabi, Mehrab Sayadi, Alireza Sadeghi, Negar Azarpira

**Affiliations:** ^1^ Transplant Research Center, Shiraz University of Medical Sciences, Shiraz, Iran; ^2^ Cardiovascular research center, Shiraz University of Medical Sciences, Shiraz, Iran; ^3^ Gastroenterohepatology Research Center, Shiraz University of Medical Sciences, Shiraz, Iran

**Keywords:** melatonin, infertility, rodents, oxidative stress, environmental pollutants, heavy metals

## Abstract

**Background:**

Environmental pollution and infertility are two modern global challenges that agonize personal and public health. The causal relationship between these two deserves scientific efforts to intervene. It is believed that melatonin maintains antioxidant properties and may be utilized to protect the testicular tissue from oxidant effects caused by toxic materials.

**Methods:**

A systematic literature search was conducted in PubMed, Scopus, and Web of Science to identify the animal trial studies that evaluated melatonin therapy’s effects on rodents’ testicular tissue against oxidative stress caused by heavy metal and non-heavy metal environmental pollutants. Data were pooled, and standardized mean difference and 95% confidence intervals were estimated using the random-effect model. Also, the risk of bias was assessed using the Systematic Review Centre for Laboratory animal Experimentation (SYRCLE) tool. (PROSPERO: CRD42022369872)

**Results:**

Out of 10039 records, 38 studies were eligible for the review, of which 31 were included in the meta-analysis. Most of them showed beneficial effects of melatonin therapy on testicular tissue histopathology. [20 toxic materials were evaluated in this review, including arsenic, lead, hexavalent chromium, cadmium, potassium dichromate, sodium fluoride, cigarette smoke, formaldehyde, carbon tetrachloride (CCl4), 2-Bromopropane, bisphenol A, thioacetamide, bisphenol S, ochratoxin A, nicotine, diazinon, Bis(2-ethylhexyl) phthalate (DEHP), Chlorpyrifos (CPF), nonylphenol, and acetamiprid.] The pooled results showed that melatonin therapy increased sperm count, motility, viability and body and testicular weights, germinal epithelial height, Johnsen's biopsy score, epididymis weight, seminiferous tubular diameter, serum testosterone, and luteinizing hormone levels, testicular tissue Malondialdehyde, glutathione peroxidase, superoxide dismutase, and glutathione levels. On the other hand, abnormal sperm morphology, apoptotic index, and testicular tissue nitric oxide were lower in the melatonin therapy arms. The included studies presented a high risk of bias in most SYRCLE domains.

**Conclusion:**

In conclusion, our study demonstrated amelioration of testicular histopathological characteristics, reproductive hormonal panel, and tissue markers of oxidative stress. Melatonin deserves scientific attention as a potential therapeutic agent for male infertility.

**Systematic review registration:**

https://www.crd.york.ac.uk/PROSPERO, identifier CRD42022369872.

## Graphical Abstract

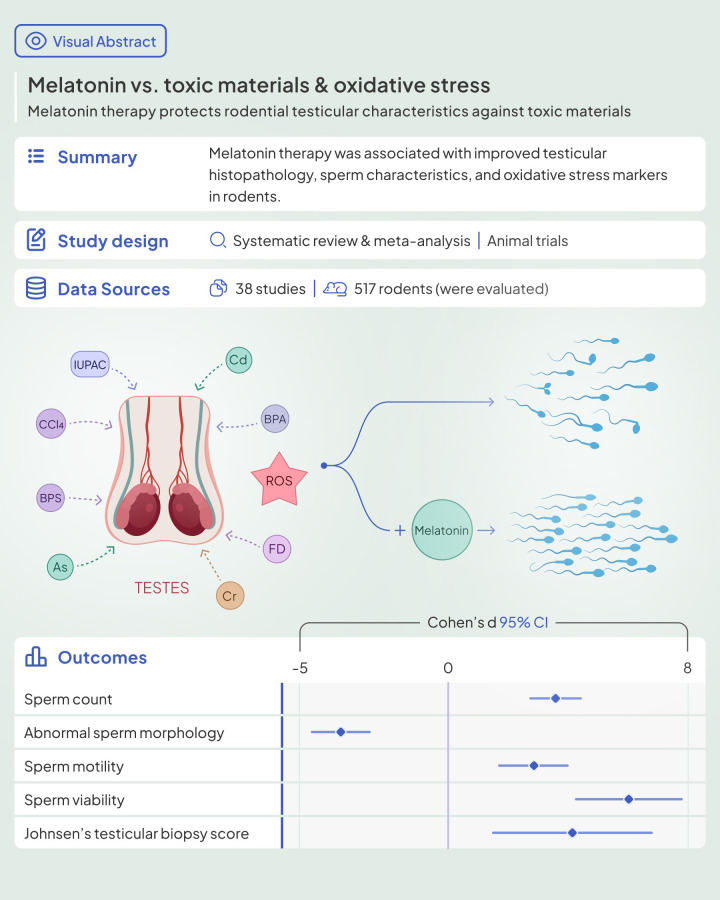

## 1 Introduction

Infertility is a universal public health issue with a dramatically increasing prevalence in recent decades (1). About 48 million couples worldwide suffer from fecundity problems (2), of which half have been implicated by male factors (3). Male infertility may result from various factors such as genetic, epigenetic, physical injuries, drugs, and environmental pollutants. Among these, pesticides, plasticizers, refrigerants, dry cleaners, and heavy metals are of great interest as they are widely used in industry. Environmental pollutants impair the function of male fecundity by altering hormonal, molecular, and histological characteristics. These pollutants mostly affect biomechanics through oxidative stress and increasing free radicals causing a shift in the equilibrium between the production of free-radical species and the antioxidant defense system in male reproductive cells (4, 5).

Reactive oxygen species (ROS) are highly reactive molecules generated by cellular metabolism. A physiological level of ROS is essential for the proper development of spermatozoa, including their production, maturation, morphological reshaping, and fertilization process (6). Redox reactions serve as cofactors for the spermatozoa maturation, free radicals also stimulate intracellular pathways resulting acrosome reaction, capacitation, motility, and condensation of chromatin (7–9). ROS concentration increases in excessive exposure to environmental pollutants (10); This excessive ROS overwhelms the cellular antioxidant defense system and accelerates oxidative stress (11). The elevated ROS attacks multiple cellular macromolecules leading to DNA damage, lipid peroxidation, and protein misfolding, resulting in mitochondrial dysfunction and sperms’ structural integrity impairment (12–16). The damage worsens by changes in the apoptotic index, cell vacuolization, and diminished capacity to proliferate, followed by a reduction in sperm viability and count (17, 18). Among the different cell types, spermatozoa are highly vulnerable to oxidation due to the abundance of unsaturated fatty acids in the membrane, lack of proper DNA repair mechanisms, and the absence of cytoplasmic antioxidant enzymes, with concomitant negative consequences on sperm quality (19–23).

Antioxidants have gained attention for their role in infertility (24–27). So far, various supplements with antioxidant capabilities, such as ginger, vitamin C, and vitamin E, have been shown to improve hormonal and histological parameters related to male reproduction (28–30). Melatonin also has exhibited potent protective activities against oxidative stress-induced testicular cell damage (31). Melatonin synthetic enzymes and melatonin membrane receptors are identified in testicular cells, indicating the importance of the melatonergic system in male reproduction. It also directly affects testosterone production from Leydig cells (32). Melatonin has recently gained scientific interest due to its protection against oxidative stress-induced testicular cell damage (33, 34). It protects testicular cells from elevated ROS through anti-apoptotic and antioxidant activities (34).

Despite the wealth of evidence, consensus and structured gathering of evidence are still needed. This systematic review and meta-analysis aims to congregate the evidence on melatonin as a protective agent against rodential testicular damages caused by environmental pollutants and toxic materials.

## 2 Material and methods

This systematic review of relevant studies was conducted according to the Preferred Reporting Items for Systematic Reviews and Meta-analyses guideline (PRISMA). The protocol is registered in the International Prospective Register of Systematic Reviews (PROSPERO: CRD42022369872). We systematically searched PubMed, Scopus, and Web of Science from January 1, 1970, until September 9, 2022, for “melatonin” and “reproductive indices” related terms (**Supplementary Material 1**). Also, we manually searched the reference list of the included papers for additional citations of interest.

### 2.1 Study selection and eligibility criteria

Firstly, duplicate records were removed automatically. All the records were uploaded to the Rayyan online tool for managing systematic reviews. Three reviewers (NDE, AS, and MAS) screened the records by title and abstract. Then, records were screened for eligibility criteria. Discrepancies were resolved with discussion. Studies were included if they fulfilled the following criteria (1): controlled animal studies (2), the subjects were rodents that were exposed to toxic environmental materials such as environmental pollutants and heavy metals to induce oxidative stress (3), at least one intervention group received melatonin regimen (4), at least one control group with similar oxidative stress that did not receive melatonin (with or without placebo), and (5) the study reported major hallmarks of testicular tissue (histopathologic, biochemical, and sperm analyses). We excluded the studies if they (1): designed as *in-vitro* and ex-vivo, (2) employed non-rodent animals, (3) studied other types of stressors such as physical, ischemic, heat, radiation, chemotherapy, and metabolic agents, (4) melatonin was administered in combination with other drugs or the study employed melatonin derivatives, (5) only evaluated healthy controls without oxidative stress, and (6) they failed to report favorable outcomes. Also, we excluded reviews, letters, and human trials.

### 2.2 Data extraction and assessment of risk of bias

Two reviewers (FN and NDE) independently extracted the data into Excel spreadsheets, and three reviewers (AS, MAS, and AM) rechecked the data for any mistakes. The following data were extracted from each study: (1) study characteristics (first author, publication year, and country), (2) population characteristics (species, age, and sample size), (3) toxic material, dose, administration route, and duration of exposure, (4) melatonin dose, duration, route, and setting of administration (before, simultaneous, or after oxidative stress), (5) sperm characteristics (count, motility, viability, and abnormal morphology), testicular parameters (height of germinal epithelium, Johnsen’s testicular biopsy score (JTBS), seminiferous tubular and luminal diameter, and apoptotic index), hormonal panel (serum testosterone, Follicle-Stimulating Hormone (FSH), Luteinizing Hormone (LH)), markers of oxidative stress (testicular tissue Superoxide dismutase (SOD), Catalase (CAT) activity, Malondialdehyde (MDA), glutathione peroxidase (GPx), glutathione (GSH), and nitric oxide (NO), and somatic characteristics (testis to body relative weight, total testis weight, epididymis weight, body weight, and body weight gain).

Two reviewers (NDE and AS) independently assessed the risk of bias using the Systematic Review Centre for Laboratory Animal Experimentation (SYRCLE) tool for animal intervention studies.

### 2.3 Data synthesis and statistical analyses

Meta-analysis was run *via* Stata 13 (College Station, TX, USA) using the DerSimonian-Laird random effect model. Standardized mean difference (SMD) was considered as the effect size for comparing the mean difference of variables between the control and intervention groups. The amount of heterogeneity in the studies was indicated by I-squared. Subgroup analyses were done where at least two studies were available in each subgroup to investigate the differences between heavy metal and non-heavy metal pollutants. Also, the forest plot was provided for each study, and pooled data publication bias was assessed by Egger’s test. In addition, sensitivity analysis was done to check for the robustness of our results.

## 3 Results

### 3.1 Search results

**Figure 1:**
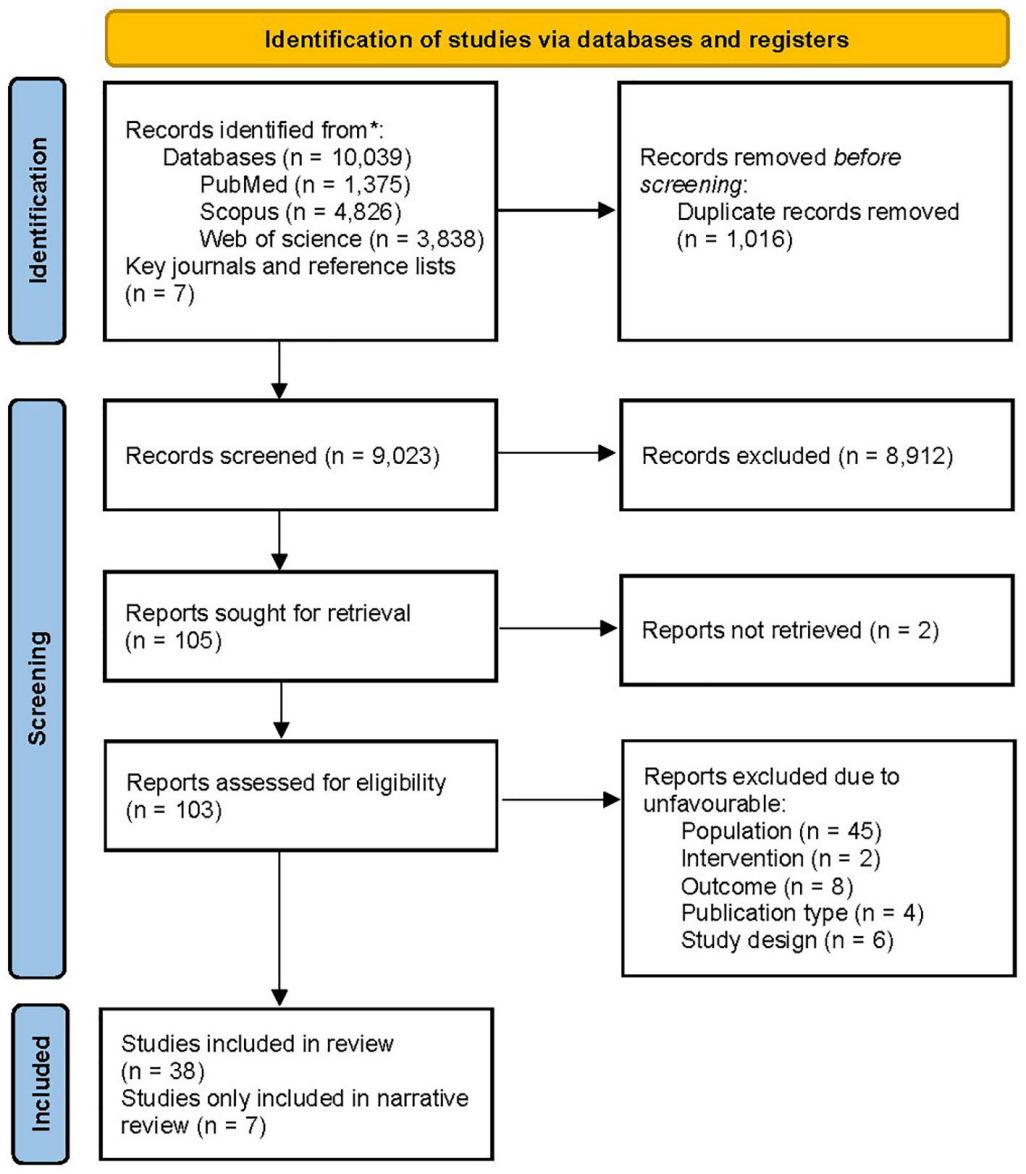
PRISMA flow chart of the literature screening. PRISMA, Preferred Reporting Items for Systematic Reviews and Meta-Analyses.

The PRISMA flow diagram of the literature search is presented in **Figure 1**. The systematic search resulted in 10,039 records while manual citation searching yielded 7 additional studies. The database searching included PubMed (n=1,375), Web of Science (n=3,838), and Scopus (n=4,826). 1,016 records were removed using automatic duplicate detection. Title and abstract screening was conducted on 9,023 records and 98 studies was sought for retrieval. With exclusion of two studies, which the full-texts were not found and our effort to communicate with the authors failed, 103 articles were assessed for eligibility. A total of 65 articles were excluded due to ineligible population (n=45), design (n=6), intervention (n=2), outcome (n=8), and publication type (n=4). Finally, a total of 38 articles were eligible for the study; among them, 7 (35–41) were only included in the narrative data synthesis.

### 3.2 Study characteristics

Studies were published between 2007 and 2022 and all were published in English except one which was in Persian (42). Mice, rats, and hamsters were the subjects of 13 (35–38, 43–51), 24 (17, 39–42, 52–70), and one (71) studies, respectively. Studies have utilized heavy metals (n= 12) including arsenic (37, 45, 67), lead (58), cadmium (42, 47, 48, 55, 68, 69), Hexavalent chromium (38), and Potassium dichromate (52) and toxic materials (n= 26) including sodium fluoride (NaF) (61), 2-Bromopropane (54), Bisphenol A (17, 43, 46, 59, 62, 63, 70), thioacetamide (56), Bisphenol S (71), ochratoxin A (57), nicotine (49), Cigarette smoke (39), diazinon (35, 36, 50), formaldehyde (40, 60), bis(2-ethylhexyl) phthalate (DEHP) (44), carbon tetrachloride (CCl4) (41, 53), Chlorpyrifos (65, 66), nonylphenol (64), and acetamiprid (51) to induce oxidative stress. To administer the stressors, oral (n= 19) (37, 44–46, 51, 52, 57–59, 63–72), intraperitoneal (n= 14, IP) (35, 36, 38, 40–42, 47–50, 54, 56, 60, 62), and subcutaneous (n= 2, SQ) (55, 63) routes were used. To administer melatonin, oral (n= 9) (37, 45, 46, 57, 58, 63, 65, 66, 68), IP (n= 24) (35, 36, 38–42, 44, 47–52, 54, 56, 59, 60, 62, 64, 67, 70–72), and SQ (n= 2) (55, 63) routes were used. Melatonin was administered prior to (n= 18, preventive) (17, 35, 36, 41, 46–48, 50, 52, 54, 56, 62, 64–66, 70–72), simultaneously (n= 6) (38, 40, 42, 45, 59, 68), and after (n= 12, therapeutic) (37, 39, 43, 49, 53, 56–58, 60, 63, 67, 69) the start of stressor. The characteristics of the included studies are summarized in the **Tables 1**, **2**.

**Table 1:** General characteristics of the included studies.

Study name	Rodent	Number of subjects (intervention, control)	Age of subject	Injury agent
Studies that employed heavy metals				
**Uygur [2013]** (67)	Rats	9, 9	6 weeks	Arsenic
**Olayaki [2018]** (58)	Rats	5, 5	NA	Lead
**Lv [2017]** (38)	Mice	6, 6	8 weeks	Hexavalent chromium
**Bustos-Obregón [2013] a** (37)	Mice	NA, NA	3 months	Arsenic
**Sobhani [2015] (Persian)** (42)	Rats	8, 8	6-8 weeks	Cadmium
**Bashandy [2021]** (52)	Rats	8, 8	3 months	Potassium dichromate (PDC)
**Bustos-Obregón [2013] b** (45)	Mice	22, 22	12 weeks	Arsenic
**Venditti [2021] b** (68)	Rats	6, 6	8 weeks	Cadmium
**Venditti [2021] a** (69)	Rats	6, 6	2 months	Cadmium
**Ji [2011]** (47)	Mice	12, 12	8 weeks	Cadmium
**Kara [2007]** (55)	Rats	12, 12	14–16 weeks	Cadmium
**Li [2015]** (48)	Mice	10, 10	8 weeks	Cadmium
Studies that employed environmental pollutants				
**Rao [2012]** (61)	Rats	15, 15	NA	Sodium Fluoride (NaF)
**Aslani [2015]** (39)	Rats	5, 5	3-4 months	Cigarette smoke
**Abd el salam [2020]** (40)	Rats	10, 10	N/A	Formaldehyde
**Wang [2018]** (41)	Rats	8, 8	N/A	Carbon tetrachloride (CCl4)
**Huang [2009]** (54)	Rats	6, 6	8 weeks	2-Bromopropane
**Kadir [2021]** (63)	Rats	6, 6	1 day	Bisphenol A
**Karabulut [2020]** (56)	Rats	7, 7	NA	Thioacetamide
**Kumar [2020]** (71)	Hamsters	6, 6	90-100 days	Bisphenol S
**Malekinejad [2014]** (57)	Rats	8, 8	8 weeks	Ochratoxin A (OTA)
**Mohammadghasemi [2018]** (49)	Mice	8, 8	10–12 weeks	Nicotine
**Sarabia [2011]** (50)	Mice	6, 6	12 weeks	Diazinon
**Othman [2014]** (17)	Rats	8, 8	8 weeks	Bisphenol A
**Ozen [2008]** (60)	Rats	7, 7	NA	Formaldehyde
**Anjum [2011]** (43)	Mice	6, 6	NA	Bisphenol A
**Bahrami [2018]** (44)	Mice	8, 8	4 weeks	Bis(2-ethylhexyl) phthalate (DEHP)
**Sarabia [2009] a** (36)	Mice	NA, NA	12 weeks	Diazinon
**Dunjić [2022]** (53)	Rats	6, 6	NA	Carbon tetrachloride (CCl4)
**Ajani [2019] (thesis)** (59)	Rats	10, 10	NA	Bisphenol A
**Umosen [2014]** (66)	Rats	6, 6	7-8 weeks	Chlorpyrifos (CPF)
**Elwakeel [2018]** (46)	Mice	6, 6	9-12 months	Bisphenol A
**Rashad [2021]** (62)	Rats	7, 7	8 weeks	Bisphenol A
**Sarabia [2009] b** (35)	Mice	NA, NA	12 weeks	Diazinon
**Tabassum [2016]** (64)	Rats	8, 8	NA	Nonylphenol
**Umosen [2012]** (65)	Rats	10, 10	NA	Chlorpyrifos (CPF)
**Wu [2013]** (70)	Rats	10, 10	8 weeks	Bisphenol A
**Zayman [2022]** (51)	Mice	6, 7	32 weeks	Acetamiprid

NA, Not Available.

**Table 2:** Details on experimental designs of each included study.

Study name	Injury agent						Melatonin							
	Name	Route	Each dose (mg/kg)	Duration		Cumulative dose (mg/kg)	Route	Each dose (mg/kg)			Duration	Cumulative dose (mg/kg)	Mode of intervention	
Studies that employed heavy metals														
**Uygur [2013]** (67)	Arsenic	Oral	5	30 days		150	IP	25			30 days	750	Therapeutic
**Olayaki [2018]** (58)	Lead	Oral	50	28 days		1400	Oral	4 and 10			2 and 4 weeks	56, 112, 140, and 280	Therapeutic
**Lv [2017]** (38)	Hexavalent chromium	IP	16.2	7 days		113.4	IP	25			7 days	175	Simultaneous
**Bustos-Obregón [2013] a** (37)	Arsenic	Oral	7	8, 32, and 66 days		58.1, 225.4, and 464.8	Oral	10			8, 32, and 66 days	80, 320, and 660	Therapeutic
**Sobhani [2015] (Persian)** (42)	Cadmium	IP	2	30 days		60	IP	10, 15, and 20			30 days	300, 450, and 600	Simultaneous
**Bashandy [2021]** (52)	Potassium dichromate (PDC)	Oral	10	56 days		560	IP	2.5 and 5			8 weeks	140 and 280	Preventive
**Bustos-Obregón [2013] b** (45)	Arsenic	Oral	7	33 days		231	Oral	10			33 days	330	Simultaneous
**Venditti [2021] b** (68)	Cadmium	Oral	50	NA		NA	Oral	NA			NA	NA	Simultaneous
**Venditti [2021] a** (69)	Cadmium	Oral	50 mg CdCl2/L	40 days		2000 mg CdCl2/L	NA	3 mg/L			40 days	120 mg/L	Therapeutic
**Ji [2011]** (47)	Cadmium	IP	2	1 day		2	IP	5			1-2 days	20	Preventive
**Kara [2007]** (55)	Cadmium	SQ	1	30 days		30	SQ	10			1 month	300	NA
**Li [2015]** (48)	Cadmium	IP	2	7 days		14	IP	10			7 days	70	Preventive
Studies that employed environmental pollutants														
**Rao [2012]** (61)	Sodium Fluoride (NaF)	Oral	10	60 days		600	IP	10			60 days	600	Preventive	
**Aslani [2015]** (39)	Cigarette smoke	Inhaled	30 minutes	3 days		90 minutes	IP	25			5 days	125	Therapeutic	
**Abd el salam [2020]** (40)	Formaldehyde	IP	10	30 days		150	IP	25			30 days	375	Simultaneous	
**Wang [2018]** (41)	Carbon tetrachloride (CCl4)	IP	8 g/kg	1 day		8 g/kg	IP	10			2 days	20	Preventive	
**Huang [2009]** (54)	2-Bromopropane	IP	1000	7 days		7000	IP	5			1 day	5	Preventive	
**Kadir [2021]** (63)	Bisphenol A	SQ and oral	25 and 50	4 and 49 days		100 and 200	SQ and oral	10			4 and 49 days	40 and 490	Therapeutic	
**Karabulut [2020]** (73)	Thioacetamide	IP	300	1 day		600	IP	10			1 and 2 day	10 and 20	Therapeutic and preventive	
**Kumar [2020]** (71)	Bisphenol S	Oral	75	28 days		2100	IP	10			28 days	140	Preventive	
**Malekinejad [2014]** (57)	Ochratoxin A (OTA)	Oral	0.2	28 days		5.6	Oral	15			28 days	420	Therapeutic	
**Mohammadghasemi [2018]** (49)	Nicotine	IP	1	30 days		3	IP	10			30 days	300	Therapeutic	
**Sarabia [2011]** (50)	Diazinon	IP	21.6 and 43.3	1 day		21.6 and 43.3	IP	10			Single dose	10	Preventive	
**Othman [2014]** (17)	Bisphenol A	NA	50	21 and 42 days		450 and 900	NA	10			3 and 6 weeks	90 and 180	Preventive	
**Ozen [2008]** (60)	Formaldehyde	IP	10	30 days		150	IP	25			30 days	375	Therapeutic	
**Anjum [2011]** (43)	Bisphenol A	NA	10	14 days		140	NA	10			14 days	140	Therapeutic	
**Bahrami [2018]** (44)	Bis(2-ethylhexyl) phthalate (DEHP)	Oral	2000	14 days		28000	IP	10			14 days	140	NA	
**Sarabia [2009] a** (36)	Diazinon	IP	21.6 and 43.3	1 day		21.6 and 43.3	IP	10			Single dose	10	Preventive	
**Dunjić [2022]** (53)	Carbon tetrachloride (CCl4)	NA	1 ml/kg	1 day		1 ml/kg	NA	50			Single dose	50	Therapeutic	
**Ajani [2019] (thesis)** (59)	Bisphenol A	Oral	10	45 days		450	IP	10			45 days	450	Simultaneous	
**Umosen [2014]** (66)	Chlorpyrifos (CPF)	Oral	8.5	28 days		238	Oral	0.5			28 days	14	Preventive	
**Elwakeel [2018]** (46)	Bisphenol A	Oral	50 and 100	48 days		900 and 1800	Oral	100			48 days	1800	Preventive	
**Rashad [2021]** (62)	Bisphenol A	IP	50	21 days		450	IP	10			21 days	90	Preventive	
**Sarabia [2009] b** (35)	Diazinon	IP	21.6 and 43.3	1 day		21.6 and 43.3	IP	10			Single dose	10	Preventive	
**Tabassum [2016]** (64)	Nonylphenol	Oral	25	45 days		1125	IP	10			45 days	450	Preventive	
**Umosen [2012]** (65)	Chlorpyrifos (CPF)	Oral	8.5	28 days		238	Oral	0.5			28 days	14	Preventive	
**Wu [2013]** (70)	Bisphenol A	Oral	200	10 days		2000	IP	10			10 days	100	Preventive	
**Zayman [2022]** (51)	Acetamiprid	Oral	25	21 days		525	IP	20			21 days	420	NA	

IP, Intraperitoneal; SQ, Subcutaneous; CdCl2, Cadmium chloride; NA, Not Available.

### 3.3 Sperm and somatic characteristics

Sperm characteristics were reported in the included studies as abnormal morphology, count, motility, and viability. Melatonin therapy significantly improved all these parameters: abnormal morphology (SMD -3.59 with 95% CI -4.60, -2.59), count (SMD 3.56 with 95% CI 2.7, 4.42), motility (SMD 2.92 with 95% CI 2.16, 3.69), and viability (SMD 5.99 with 95% CI 4.19, 7.78), all with p-values <0.001.

**Figure 2:**
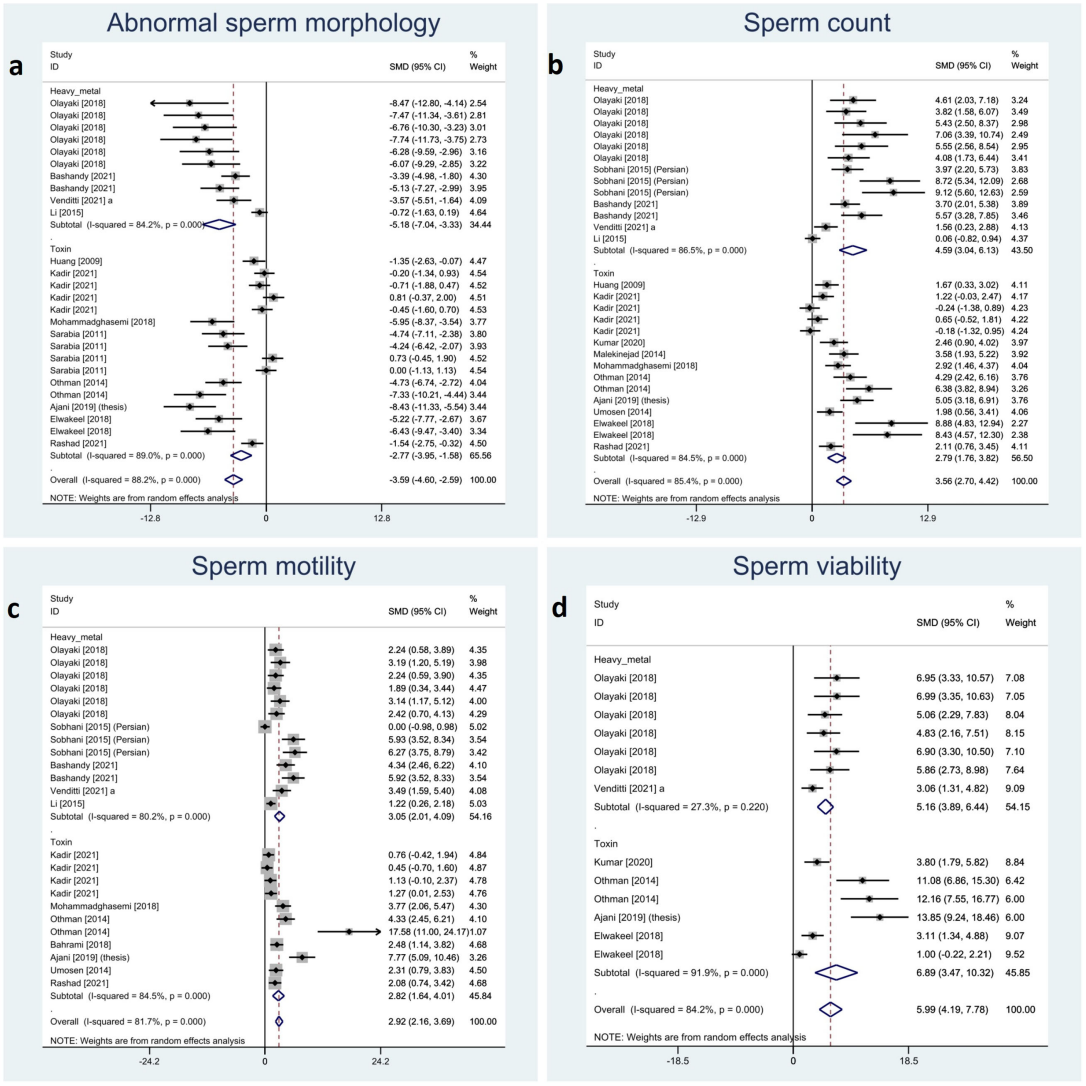
Forest plots for overall and subgroup effect measures on the impact of melatonin therapy on sperm characteristics including **(A)** abnormal sperm morphology, **(B)** count, **(C)** motility, and **(D)** viability.

Between-study heterogeneity was substantial for all these outcomes with I-squared ranging between 81% and 88% and p-values <0.001 for all outcomes. Also, Egger’s test showed statistically significant publication bias in all the outcomes with p-values <0.001. Forest plots of analyses for sperm parameter outcomes are presented in **Figure 2**.

**Figure 3:**
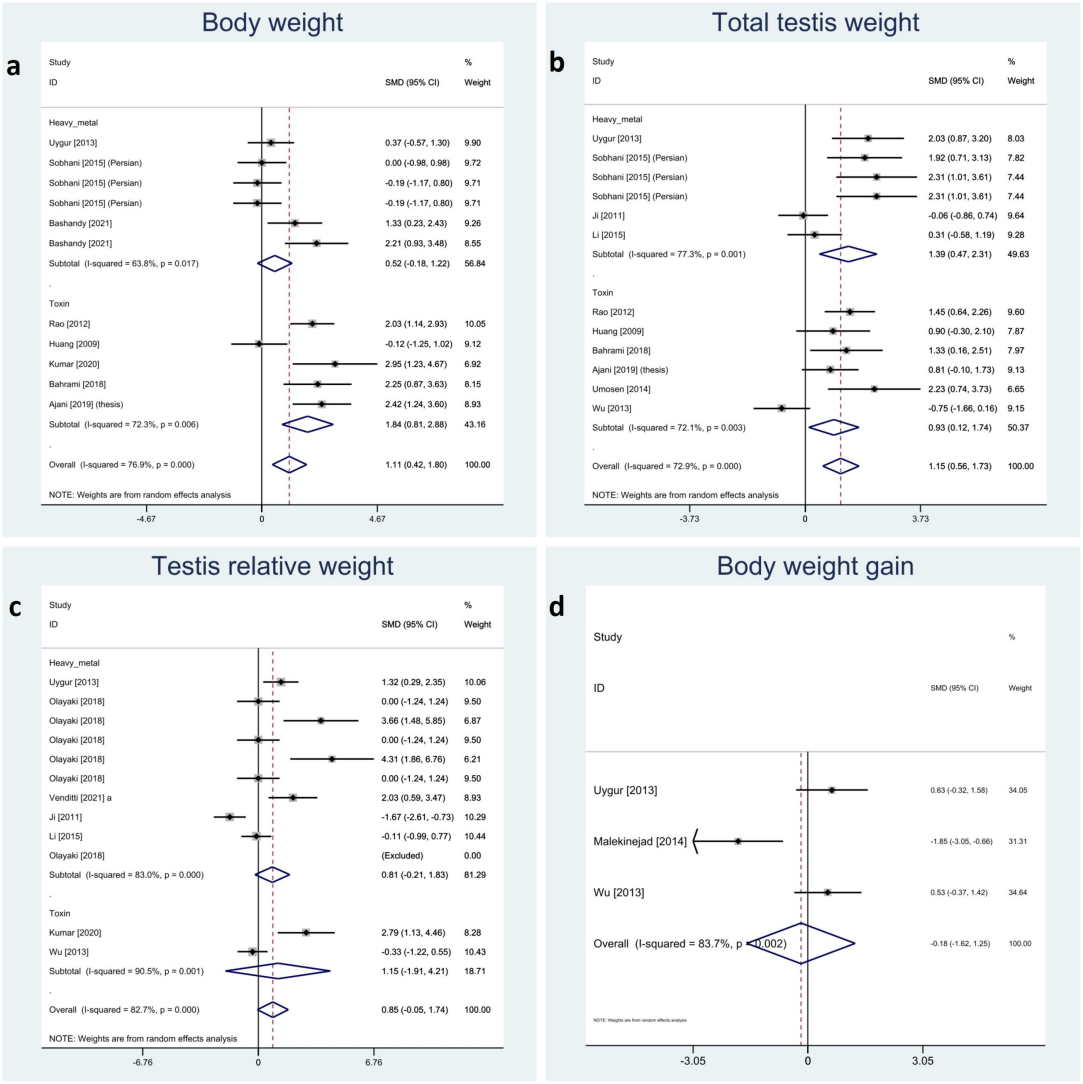
Forest plots for overall and subgroup effect measures on the impact of melatonin therapy on the **(A)** body, **(B)** total and **(C)** relative testicular weights and **(D)** body weight gain.

We categorized relative testis to body, total testicular, and body weight and body weight gain as somatic indices. The meta-analyses showed a significant impact of melatonin therapy on total testicular and body weight (SMD 1.15 with 95%CI 0.56, 1.73 and p-value <0.001 and SMD 1.11 with 95%CI 0.42, 1.80 and p-value 0.002, respectively). Although, testis to body relative weight and body weight gain were not significantly affected by melatonin therapy (SMD 0.85 with 95%CI -0.05, 1.74 and p-value 0.064 and SMD -0.18 with 95%CI -1.62, 1.25 and p-value 0.803, respectively). Investigation of between-study variation revealed substantial heterogeneity with I-squared ranging between 72% and 83% (p-values <0.001 and 0.002). Assessment of publication bias was not feasible for body weight gain due to low sample size. Egger’s test showed statistically significant publication bias in testis to body relative weight and total testicular weight (p-value 0.003 and 0.012, respectively). Forest plots and detailed results of Egger’s test for sperm parameter outcomes is presented in **Figure 3** and **Supplementary Material 2**.

### 3.4 Testicular tissue parameters

Testicular parameters were reported in the included studies as height of germinal epithelium, JTBS, tubular diameter, luminal diameter, epididymis weight, and apoptotic index. Meta-analyses on these variables showed that melatonin therapy significantly increased height in germinal epithelium (SMD 3.63 with 95% CI 2.05, 5.21 and p-value <0.001), JTBS (SMD 4.13 with 95% CI 1.44, 6.81 and p-value <0.001), tubular diameter (SMD 2.44 with 95% CI 1.41, 3.47 and p-value <0.001), and epididymis weight (SMD 1.03 with 95% CI.014, 1.93 and p-value 0.024) and decreased apoptotic index (SMD -4.07 with 95% CI -7.23, -0.91 and p-value 0.012). Although not statistically significant, melatonin therapy increased luminal diameter (SMD 0.45 with 95% CI -0.90, 1.79 and p-value 0.515).

**Figure 4:**
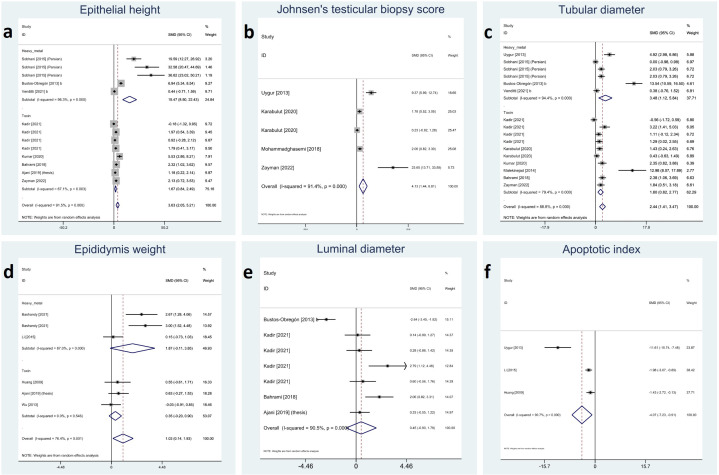
Forest plots for overall and subgroup effect measures on the impact of melatonin therapy on the parameters of testicular tissue including **(A)** epithelial height, **(B)** Johnsen’s biopsy score, **(C)** seminiferous tubular diameter, **(D)** epididymis weight, **(E)** seminiferous luminal diameter, and **(F)** apoptotic index.

Between-study heterogeneity was considerable for all these outcomes with I-squared ranging between 76% and 91% and p-values <0.001 for all outcomes. Egger’s test showed statistically significant publication bias in all the outcomes with p-values <0.001 for tubular diameter, and epithelial height and 0.011, 0.026 and 0.008 for JTBS, luminal diameter, and epididymis, respectively. Forest plots and detailed results of Egger’s test for sperm parameter outcomes are presented in **Figure 4** and **Supplementary Material 2**.

### 3.5 Reproductive hormones

**Figure 5:**
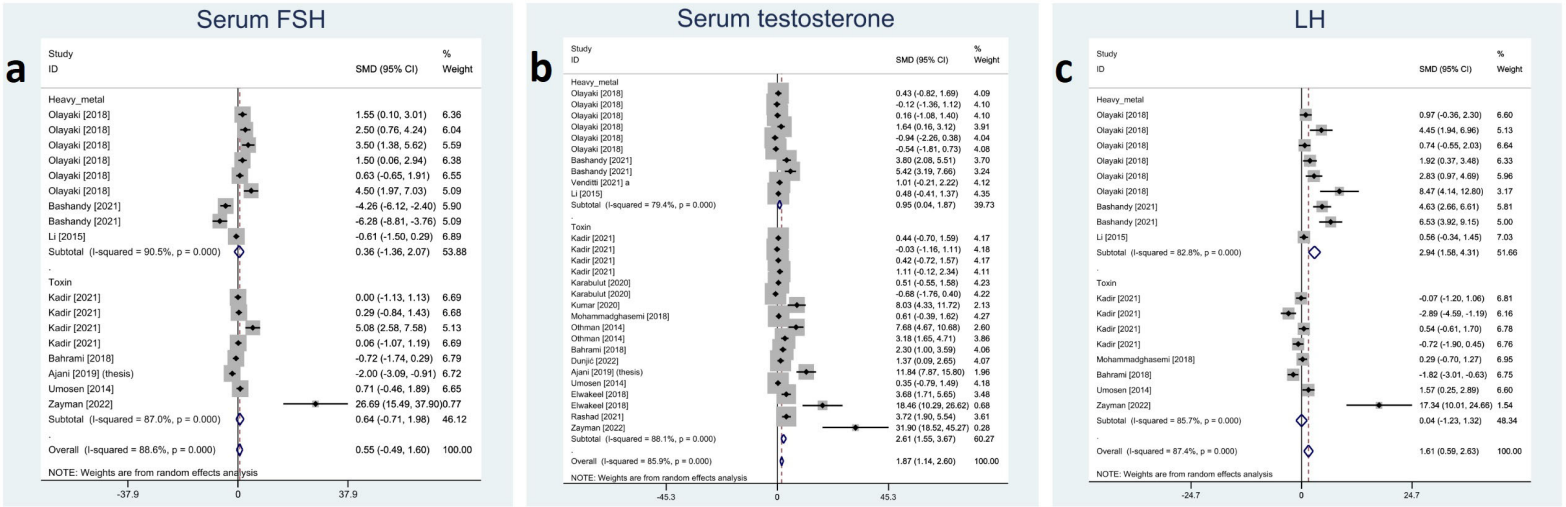
Forest plots for overall and subgroup effect measures on the impact of melatonin therapy on the serum level of reproductive hormones including **(A)** FSH, **(B)** testosterone, and **(C)** LH. FSH, Follicle-Stimulating Hormone; LH, Luteinizing Hormone.

Included studies reported serum FSH, LH, and testosterone; among them, melatonin therapy increased serum LH and testosterone significantly (SMD 1.61 with 95% CI 0.59, 2.63 and p-value 0.002 and SMD 1.87 with 95% CI 1.14, 2.60 and p-value <0.001, respectively). On the other hand, changes in serum FSH were not statistically significant (SMD 0.55 with 95% CI -0.49, 1.60 and p-value 0.299). Between-study heterogeneity was substantial or considerable for reproductive hormones I-squared ranging between 85% and 88% with p-value <0.001. Egger’s test showed significant publication bias for serum LH with p-value <0.001. Forest plots and detailed results of Egger’s test for reproductive hormones outcomes are presented in **Figure 5** and **Supplementary Material 2**.

### 3.6 Oxidative markers

**Figure 6:**
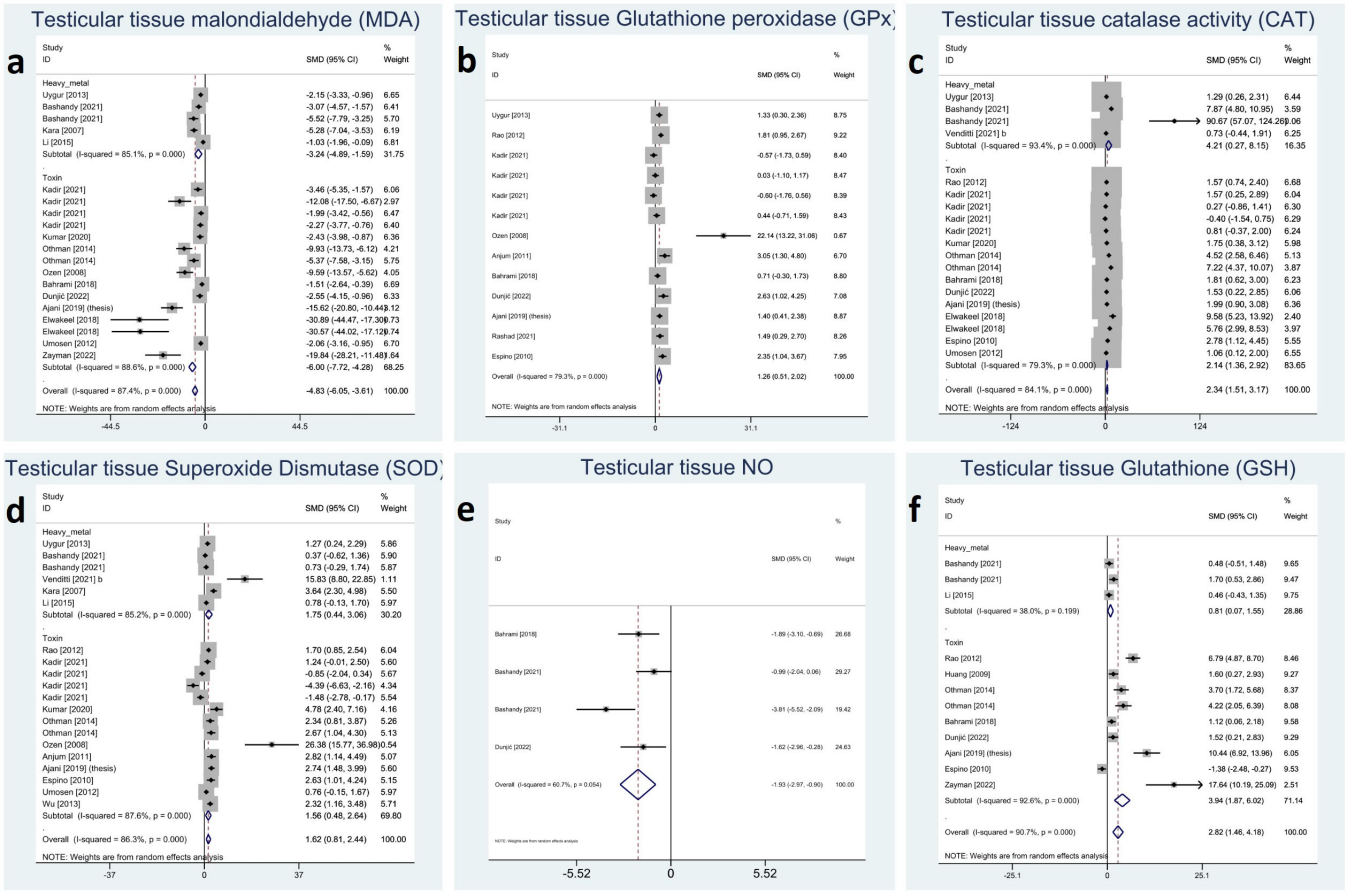
Forest plots for overall and subgroup effect measures on the impact of melatonin therapy on the testicular tissue level of oxidative stress markers including **(A)** MDA, **(B)** GPx, **(C)** CAT, **(D)** SOD, **(E)** NO, **(F)** GSH. MDA, Malondialdehyde; GPx, Glutathione Peroxidase; CAT, Catalase; SOD, Superoxide Dismutase; NO, Nitric Oxide; GSH, Glutathione.

All the reported oxidative markers showed significant changes with melatonin therapy: testicular tissue CAT (SMD 2.34 with 95%CI 1.51, 3.17), GSH (SMD 2.82 with 95%CI 1.46, 4.18), GPx (SMD 1.26 with 95%CI 0.51, 2.02), MDA (SMD -4.83 with 95%CI -6.05, -3.61), SOD (SMD 1.62 with 95%CI 0.81, 2.44), and NO (SMD -1.93 with 95%CI -2.97, -0.90) with all p-values <0.001. Between-study heterogeneity was substantial to considerable I-squared ranging between 60% to 90% with all p-values <0.001 except for NO (p-value 0.054). Using Egger’s regression model, all these outcomes suffered from publication bias (p-values <0.001) except GPx (p-value 0.992). Forest plots and detailed results of Egger’s test for sperm parameter outcomes is presented in **Figure 6** **Supplementary Material 2**.

### 3.7 Sensitivity analyses and risk of bias assessment

Sensitivity analyses were done with omitting one study each time to investigate robustness of our results. The leave-one-out plots are provided in the **Supplementary Materials 3**. After removing studies from the analyses individually, none substantially affected the pooled SMD estimates in the study.

**Figure 7:**
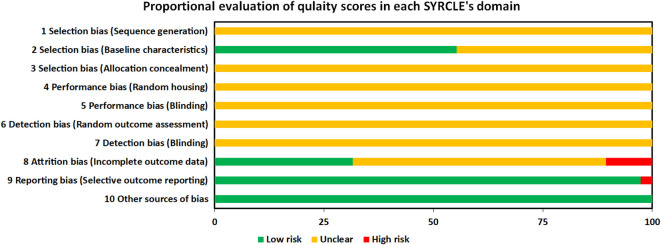
Proportional evaluation of quality score in each SYRCLE’s domain. SYRCLE, Systematic Review Centre for Laboratory Animal Experimentation.

For each domain, studies scored 1 if they were assessed as low risk. Studies scored between 2 and 4 for risk of bias assessment by SYRCKLE checklist. All the studies were labeled as unclear risk on random sequence generation, allocation concealment, random housing, blinding of investigators and outcome assessors, and random outcome assessment. For other sources of bias, all the studies were assessed as low risk. 21, 12, and 37 studies were labeled as low risk on baseline characteristics, incomplete outcome data, and selective outcome reporting, respectively. All the details are presented in the **Figure 7** and **Supplementary Material 4**.

### 3.8 Subgroup analyses

To investigate between study heterogeneity, subgroup analyses were done by categorizing the stressors as heavy metals and non-heavy metals. Abnormal sperm morphology, body and epididymis weight, epithelial height, serum LH, FSH, testosterone, sperm count, motility, viability, CAT, GSH, GPx, SOD, MDA, total and relative testicular weights, and tubular diameter were eligible for subgroup analysis. There was no significant difference between the protective effect of melatonin therapy against heavy metals and other toxins (non-heavy metals). Nonetheless, this method failed to reduce the heterogeneity within subgroups. Whenever feasible, subgroup analyses are demonstrated in the **Figures 1**-**6**.

## 4 Discussion

To the best of our knowledge, this is the first systematic review and meta-analysis examining how melatonin intake protects rodents’ male reproductive system in exposure to environmental pollutants. Environmental toxins, as potent oxidative stressors, damage male infertility by causing an imbalance between the cells’ free radical levels and the antioxidant defensive system. The following sections have gone through melatonin’s potential effects and related mechanisms to investigate its protective role on the male reproductive system.

### 4.1 Hormone parameters

The testosterone hormone mainly controls the spermatogenesis process in Sertoli cells, and LH regulates testosterone synthesis in Leydig cells (74, 75). As it is revealed by our data, melatonin intake increases serum testosterone and LH levels in male rodents injured by toxic components. These findings can be interpreted by antioxidants’ effects on reproductive hormones previously reported in reviews by Vecchio et al. and Banihani (28, 76).

Spermatogenesis, a process carried out in Sertoli cells in the testes, is mainly under testosterone control (74). Testosterone is synthesized in Leydig cells and is regulated by LH (75). Environmental pollutants may exert their effect as an endocrine disruption chemical in addition to their anti-oxidant effect (77). Some of the substances included in our study, such as Bisphenol A, Arsenic, and Cadmium, have an endocrine-disrupting effect (77, 78). Arsenic, for example, may interfere with gonadotropins’ function by suppressing their release and decreasing the transcription of androgen receptors, besides arsenic especially affects testosterone by decreasing its synthesis (79–81). Moreover, environmental pollutants by the accumulation of ROS could be accompanied by an over generation of reactive nitrogen species such as NO (82). High levels of ROS and NO generation in the testicles decrease the expression of biosynthetic enzymes, i.e., suppressing the steroidogenic acute regulatory protein (StAR) and cytochrome P450 side chain cleavage in Leydig cells (83). These cause a decrease in testosterone secretion, which is the primary hormone needed for optimal spermatogenesis (84).

Although it remains controversial, melatonin’s effect is likely to be reducing on serum testosterone levels in preclinical studies (32, 85–90). Melatonin acts directly on Leydig cells to reduce steroidogenesis and spermatogenic activity in the testes (86, 91). In our study, melatonin showed protective properties and relatively prevented the toxic effects of stressors on rodents’ serum testosterone levels in treatment arms. This effect can be explained by the protective effect of melatonin on Leydig cells against oxidative stress, increased NO, and pro-inflammatory factors (92). However, conducting more meticulous investigations in this regard is needed.

### 4.2 Oxidative stress parameters

Antioxidant defense system plays a crucial role in cells responding to environmental stresses (93). Numerous antioxidant responses are involved in antioxidant mechanisms. These responses include both non-enzymatic molecules (such as GSH) and enzymes (such as CAT, SOD, and GPx) (94). This system defends tissues and cells by scavenging free radicals against oxidative stress-related harm (72); however, it is not completely immune to free radicals (65).

As suggested by the results of this analysis, melatonin has been demonstrated to be generally essential in buffering oxidative stress. Regarding the effect of melatonin on MDA, GSH, and GPx levels, these results agree with recent meta-analyses conducted by Morvaridzade et al. and Sumsuzzman et al. (94, 95).

Environmental hazard components activate oxidative stress in testicular cells, causing damage to macromolecules involving membranes’ lipids. The testicular tissue MDA and NO levels increase by lipid peroxidation and endothelial damage, respectively. These environmental stressors also harm the pathways essential to GSH, CAT, and SOD synthesis as members of the antioxidant defense system. These changes in oxidative markers can be justified by ROS activity. ROS directly damages the macromolecules necessary for antioxidant production and overwhelms its capacity.

Melatonin plays its role by eliminating free and lipid peroxyl radicals before they act to damage macromolecules and membrane lipids (96, 97). Furthermore, it can improve CAT, GPx, SOD, and GSH expression and activity, possibly by interacting with nuclear or membrane receptors (98). Moreover, melatonin works complementary with CAT and GPx to keep the steady-state levels of intracellular H2O2, a more destructive form of free radicals with a longer half-life (96).

There are inconsistencies between our results and Sumsuzzman et al. reports regarding CAT and SOD levels (95), which are probably due to the varying types and numbers of melatonin receptors, bioavailability and concentration in different tissues, and the insufficient number of studies to support the results. Nevertheless, this concept remains controversial.

### 4.3 Sperm and somatic parameters

The results of this meta-analysis shows that melatonin significantly improves sperm parameters, including sperm count, viability, motility, and morphology. These findings align with the previous reviews regarding the ameliorating effects of antioxidants on semen qualities (28). Likewise, a systematic review by Wang et al. revealed that antioxidant treatment after varicocelectomy could significantly enhance the quality of sperm parameters (29).

In addition, findings from this systematic review and meta-analysis confirm that melatonin intake makes a marked enhancement in testicle tissue parameters, including histo-architecture, seminiferous tubular diameter, epithelial height, epididymis and total testis weight, and JTBS. In this regard, our data align with another systematic review by Tatar et al. showing the protective role of antioxidants on the weights of testes and epididymis (30).

Normal spermatogenesis is a specific determinant of semen quality (99). Toxic pollutants affect spermatogenesis by diminishing the cellular ability to proliferate and altering the apoptotic index (100). Elevated apoptosis causes a decrease in cell viability and count. Also, pollutants affect sperm motility by disturbing the function of proteins serving sperm movement as well as deterioration of the mitochondrial function to support sperm’s motion energy. Lowered testosterone levels, poor sperm quality, vacuolization in seminiferous tubules, disordered germinal epithelium, and high apoptotic index cause testicular dysfunction, leading to testicular atrophy and weight loss. As demonstrated by our data, melatonin decreases the apoptotic index, which can be justified by the free radical scavenging characteristics of melatonin. As a direct and indirect free radical scavenger, melatonin protects testis tissue/cells from dysfunctions and abnormal apoptosis.

Despite the effects of melatonin on the apoptosis index, germ cell maturation, and testosterone levels, the factors that total testis weight depends on, we did not observe any correlations in the relative testis weight. This might be due to a simultaneous modulation of body weight in melatonin-treated individuals.

The adverse effect of environmental pollutants on body weight is probably associated with their action as enzymatic toxins, eventually leading to disruption in metabolic processes that could be well modulated by melatonin administration. In this review, melatonin intervention is shown to be essential in buffering body weight against toxicity damage. This is in accordance with two other systematic reviews by Mostafavi et al. and Loloei et al. (101, 102). This observation is further supported by an earlier meta-analysis by Delpino et al., suggesting that supplemental melatonin highlighted a considerable decline in body weight after individuals experienced obesity (103).

## 5 Conclusions and future research directions

Melatonin had beneficial protective effects against oxidative stress caused by toxic materials in rodent animal models. Although included studies crucially suffered from low quality and methodological heterogeneity. Melatonin and stressor agents’ dose and duration of administration, rodents’ characteristics, and assessment strategies varied significantly across the studies. For more literature consolidation, meticulous future studies with less difference in methodology are needed.

## Data availability statement

The original contributions presented in the study are included in the article/**Supplementary Material**. Further inquiries can be directed to the corresponding author.

## Author contributions

NA and NDE conceptualized the study. AS and NDE designed the study. NE and AS searched databases. NDE and AS screened the records. NDE, FN, MS, AM, and AS extracted the data. NDE and AS performed quality assessment. AS and MS performed meta-analysis. NDE, AS, and SP provided the draft of the manuscript. NA supervised the work. All authors contributed to the article and approved the final version. AS and NDE have contributed equally to this work and share first authorship. All authors contributed to the article and approved the submitted version.
