# Supplementary material for: Protective effects of melatonin against the toxic effects of environmental pollutants and heavy metals on testicular tissue: A systematic review and meta-analysis of animal studies
Source: Front Endocrinol (Lausanne). 2023 Jan 30;14:1119553. doi: 10.3389/fendo.2023.1119553 (PMC9922902; doi:10.3389/fendo.2023.1119553)
Supplement: Supplementary file 2 [file Table_2.docx]

# Supplementary material 1

Search strategy

## Keywords

| Melatonin | Testicular function | | Mesh terms |
| --- | --- | --- | --- |
| Melatonin | "sertoli Cell" | Sterility | Infertility |
| “N-acetyl-5-methoxy tryptamine” | seminal | Subfertility | Fertility |
| “n acetyl 5 methoxytryptamine” | fertil* | “Sub-Fertility” | Fertility Agents, Male |
| "Mela-T" | epididymis | Aspermia | Testis |
| Melatol | "Vas deferens" | Fecundability | Epididymis |
| Melatonex | testes | Fecundity | Vas deferens |
| Melovine | testis | Subfecundity | Spermatogenesis |
| Regulin | testicular | Testicles | Testosterone |
| “Night NEXT Rest” | sterility | Testicle | Seminiferous Tubules |
| Circadin | sperm | “Ductus Deferens” | Seminiferous Epithelium |
|  | spermat* | Fertility AND Agents AND Male | Spermatozoa |
|  | Semen | Spermiogenesis | Semen Analysis |
|  | reproduction | Semen AND Analysis | Genitalia, Male |
|  | preconception | “17-beta-Hydroxy-4-Androsten-3-one” | Melatonin |
|  | testosterone | “17 beta Hydroxy 4 Androsten 3 one” |  |
|  | "leydig cell" | "Seminiferous Tubule" |  |
|  | seminiferous | "Seminiferous Tubules" |  |
|  | oligospermia | Seminiferous AND epithelium |  |
|  | azoospermia | Seminiferous AND epitheliums |  |
|  | astenozoospermia | Semen AND Analyses |  |
|  | infertil* | Semen AND Quality |  |
|  | Hypospermatogenes* | Sperm AND Quality |  |
|  | Oligoasthenoteratozoospermia* | Semen AND Qualities |  |
|  | Oligozoospermia | Sperm AND Qualities |  |
|  | Genital* AND Male |  |  |
|  | Reproductive AND Male |  |  |

## PubMed

(Melatonin[tiab] OR "N-acetyl-5-methoxy tryptamine"[tiab] OR "n acetyl 5 methoxytryptamine"[tiab] OR "Mela-T"[tiab] OR Melatol[tiab] OR Melatonex[tiab] OR Melovine[tiab] OR Regulin[tiab] OR "Night NEXT Rest"[tiab] OR Circadin[tiab]) AND ("sertoli Cell"[tiab] OR (sertoli[tiab] AND cell*[tiab]) OR seminal[tiab] OR fertil*[tiab] OR epididymis[tiab] OR "vas deferens"[tiab] OR (vas[tiab] AND deferens[tiab]) OR testes[tiab] OR testis[tiab] OR testicular [tiab] OR sterility[tiab] OR sperm*[tiab] OR Semen[tiab] OR reproduction[tiab] OR preconception[tiab] OR testosterone[tiab] OR "leydig cell"[tiab] OR (leydig[tiab] AND cell*[tiab]) OR "Seminiferous Tubule"[tiab] OR "Seminiferous epithelium"[tiab] OR (Seminiferous[tiab] AND Tubule*[tiab]) OR (Seminiferous[tiab] AND epithelium*[tiab]) OR oligospermia[tiab] OR hypospermatogenes*[tiab] OR Oligoasthenoteratozoospermia*[tiab] OR Oligozoospermia[tiab] OR azoospermia[tiab] OR astenozoospermia[tiab] OR infertil*[tiab] OR subfertility[tiab] OR "Sub-fertility"[tiab] OR fertil*[tiab] OR fecundability[tiab] OR fecundity[tiab] OR subfecundity[tiab] OR Aspermia[tiab] OR testicle*[tiab] OR (Ductus[tiab] AND Deferens[tiab]) OR "Ductus Deferens"[tiab] OR ("male fertility"[tiab] AND Agents[tiab]) OR "17-beta-Hydroxy-4-Androsten-3-one"[tiab] OR "17 beta Hydroxy 4 Androsten 3 one"[tiab] OR (semen[tiab] AND analys*[tiab]) OR (semen[tiab] AND qualit*[tiab]) OR (sperm[tiab] AND qualit*[tiab]) OR (Genital*[tiab] AND Male[tiab]) OR (Reproducti*[tiab] AND Male[tiab])) OR ((Melatonin[Mesh]) AND ((“Infertility”[Mesh]) OR (“Fertility”[Mesh]) OR (“Fertility Agents, Male”[Mesh]) OR (“Testis”[Mesh]) OR (“Epididymis”[Mesh]) OR (“Vas deferens”[Mesh]) OR (“Spermatogenesis”[Mesh]) OR (“Testosterone”[Mesh]) OR (“Seminiferous Tubules”[Mesh]) OR (“Seminiferous Epithelium”[Mesh]) OR (“Spermatozoa”[Mesh]) OR (“Semen Analysis”[Mesh]) OR (“Genitalia, Male”[Mesh])) AND (1965/1/1:2022/9/9[dp])

**Results: 1375**

## Scopus

TITLE-ABS-KEY(Melatonin OR "N-acetyl-5-methoxy tryptamine" OR "n acetyl 5 methoxytryptamine" OR "Mela-T" OR Melatol OR Melatonex OR Melovine OR Regulin OR "Night NEXT Rest" OR Circadin) AND TITLE-ABS-KEY("sertoli Cell" OR (sertoli AND cell*) OR seminal OR fertil* OR epididymis OR "vas deferens" OR (vas AND deferens) OR testes OR testis OR testicular OR sterility OR sperm* OR Semen OR reproduction OR preconception OR testosterone OR "leydig cell" OR (leydig AND cell*) OR "Seminiferous Tubule" OR "Seminiferous epithelium" OR (Seminiferous AND Tubule*) OR (Seminiferous AND epithelium*) OR oligospermia OR hypospermatogenes* OR Oligoasthenoteratozoospermia* OR Oligozoospermia OR azoospermia OR astenozoospermia OR infertil* OR subfertility OR "Sub-fertility" OR fertil* OR fecundability OR fecundity OR subfecundity OR Aspermia OR testicle* OR (Ductus AND Deferens) OR "Ductus Deferens" OR ("male fertility" AND Agents) OR "17-beta-Hydroxy-4-Androsten-3-one" OR "17 beta Hydroxy 4 Androsten 3 one" OR (semen AND analys*) OR (semen AND qualit*) OR (sperm AND qualit*) OR (Genital* AND Male) OR (Reproducti* AND Male)) AND PUBYEAR > 1965 AND PUBYEAR < 2023

**Results: 4826**

## Web of science

TS=(Melatonin OR "N-acetyl-5-methoxy tryptamine" OR "n acetyl 5 methoxytryptamine" OR "Mela-T" OR Melatol OR Melatonex OR Melovine OR Regulin OR "Night NEXT Rest" OR Circadin) AND TS=("sertoli Cell" OR (sertoli AND cell*) OR seminal OR fertil* OR epididymis OR "vas deferens" OR (vas AND deferens) OR testes OR testis OR testicular OR sterility OR sperm* OR Semen OR reproduction OR preconception OR testosterone OR "leydig cell" OR (leydig AND cell*) OR "Seminiferous Tubule" OR "Seminiferous epithelium" OR (Seminiferous AND Tubule*) OR (Seminiferous AND epithelium*) OR oligospermia OR hypospermatogenes* OR Oligoasthenoteratozoospermia* OR Oligozoospermia OR azoospermia OR astenozoospermia OR infertil* OR subfertility OR "Sub-fertility" OR fertil* OR fecundability OR fecundity OR subfecundity OR Aspermia OR testicle* OR (Ductus AND Deferens) OR "Ductus Deferens" OR ("male fertility" AND Agents) OR "17-beta-Hydroxy-4-Androsten-3-one" OR "17 beta Hydroxy 4 Androsten 3 one" OR (semen AND analys*) OR (semen AND qualit*) OR (sperm AND qualit*) OR (Genital* AND Male) OR (Reproducti* AND Male)) AND DOP=(1965-01-01/2022-09-09)

**Results: 3838**

# Supplementary material 2

Sensitivity analyses


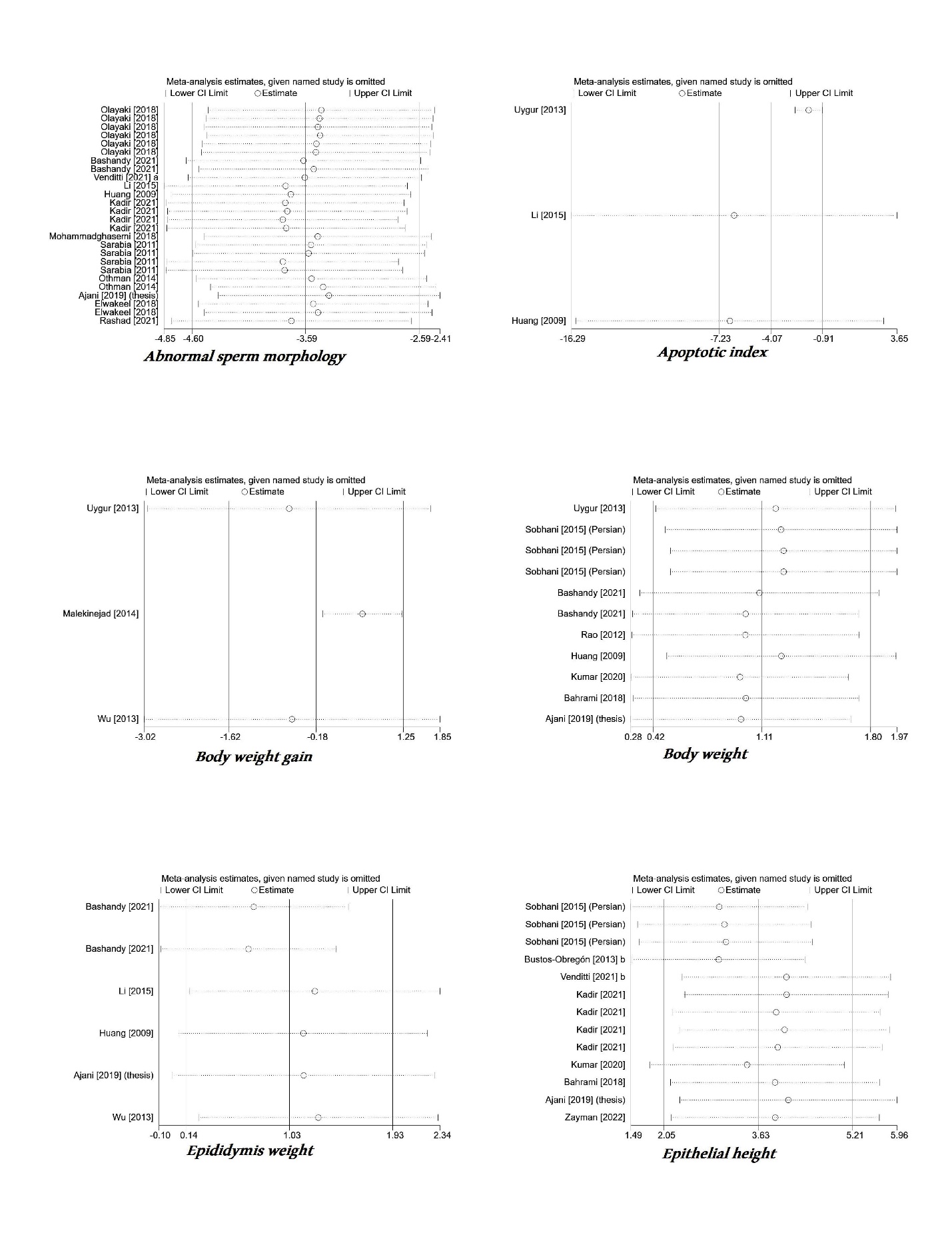

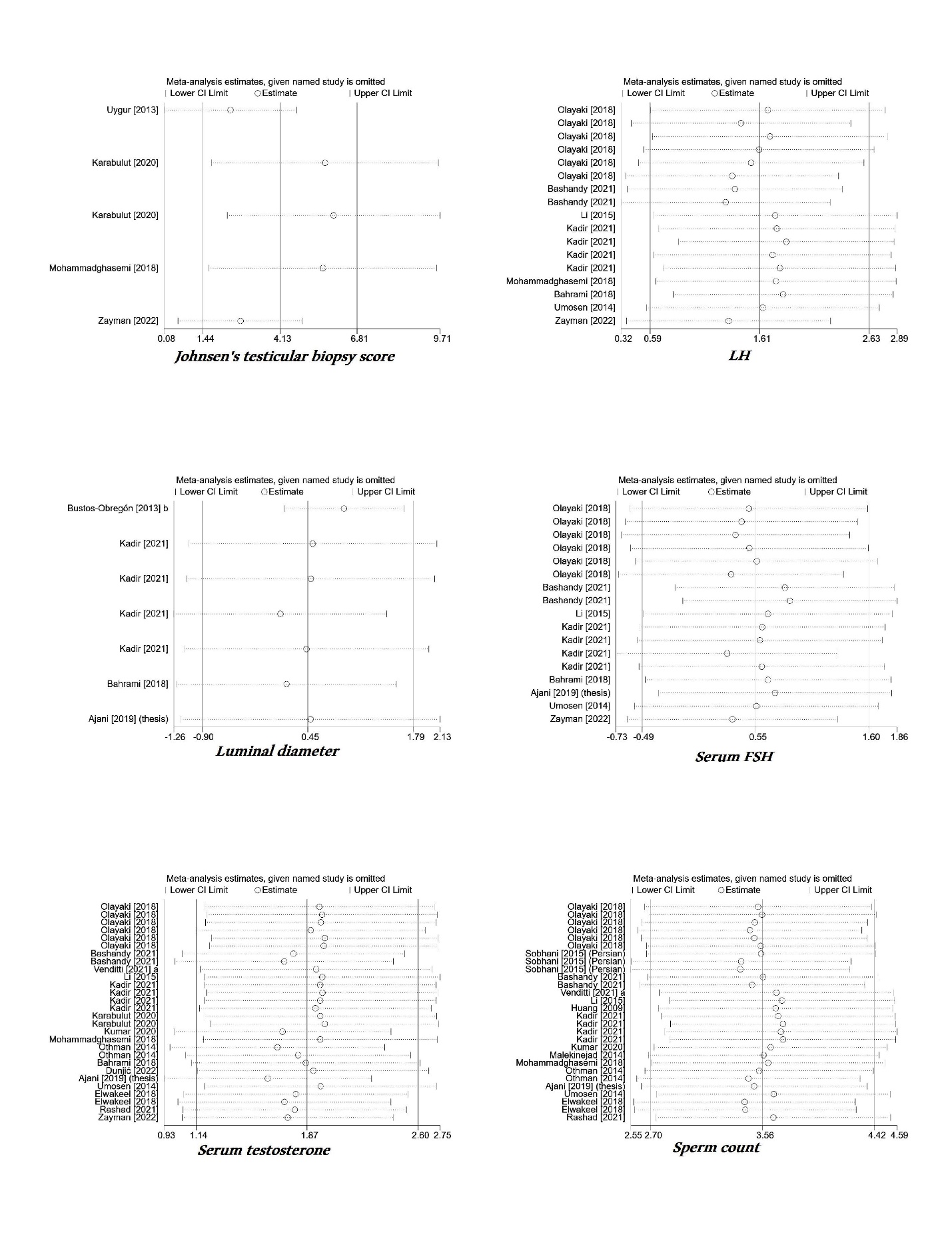

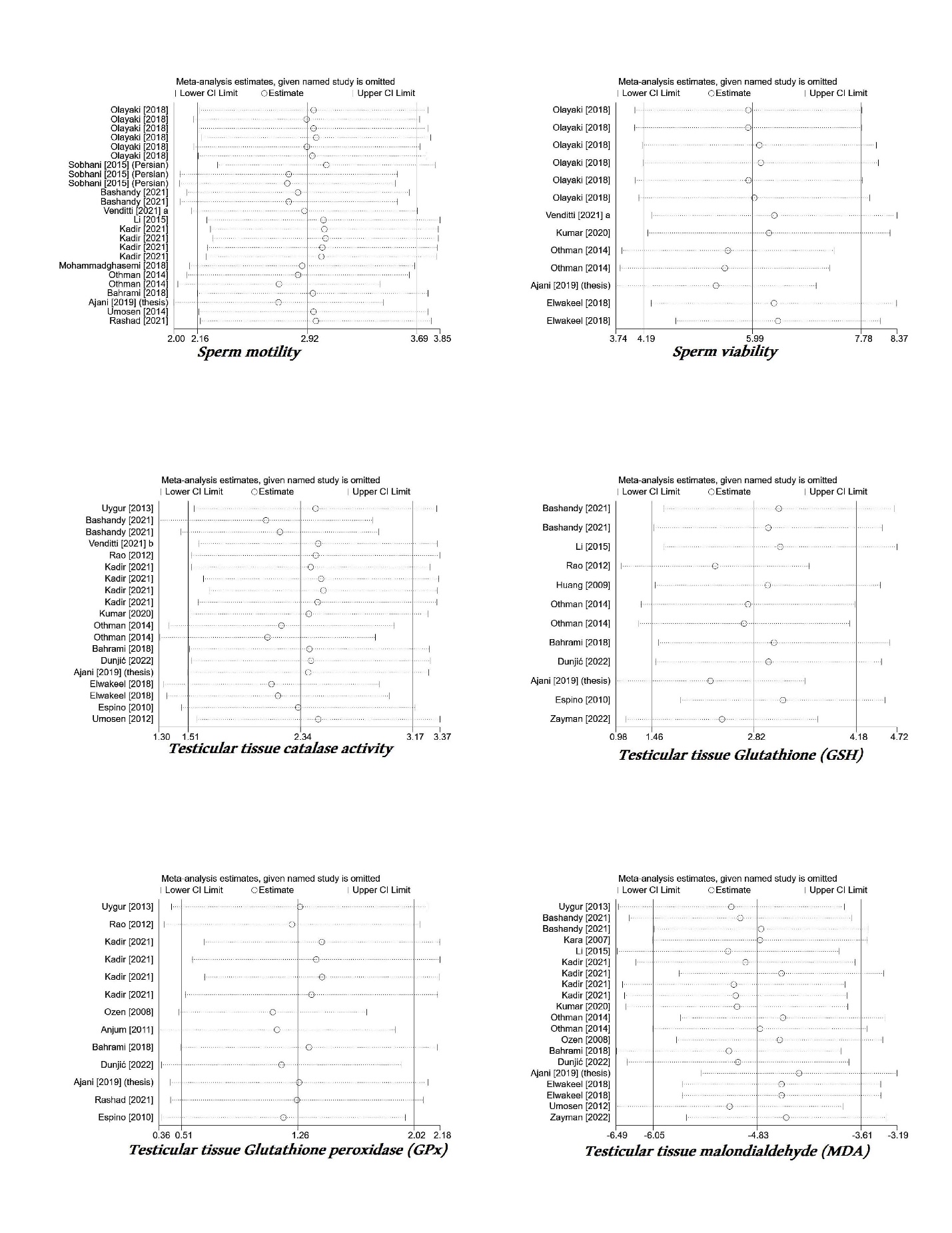

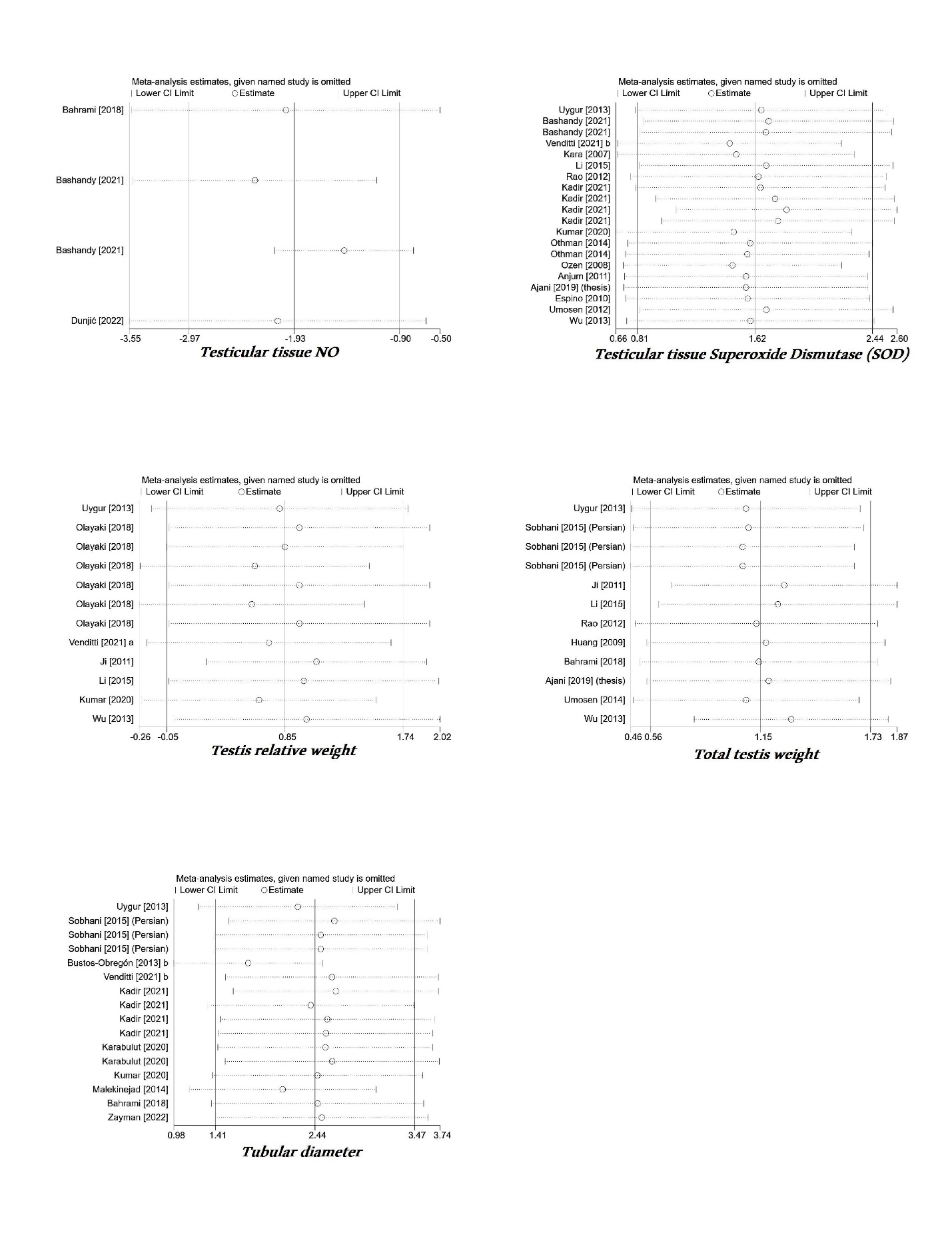


# Supplementary material 3


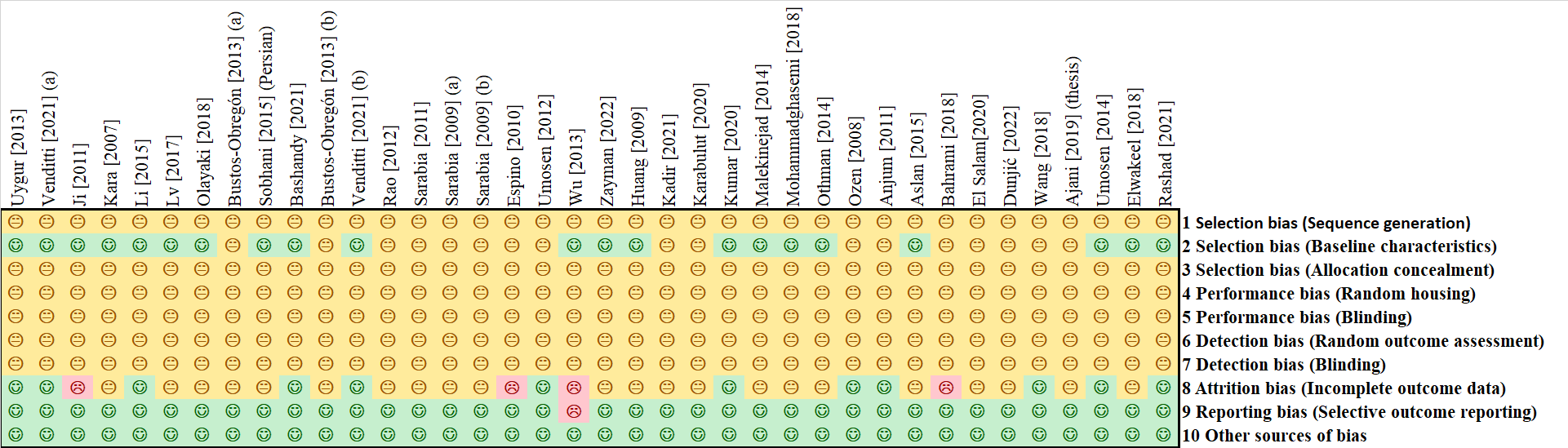


**Supplementary Material 3.** Results of risk of bias assessment with SYRCLE tool for each study. SYRCLE, Systematic Review Centre for Laboratory Animal Experimentation.
